# Spin–Orbit Torque-Driven Perpendicular Magnetization Switching for Artificial Synapses in Co/Ho Multilayer Systems

**DOI:** 10.3390/nano16040243

**Published:** 2026-02-13

**Authors:** Shaomin Li, Yidan Wei, Yuanyuan Chen, Kangyue Qu, Pingping Yu, Yanfeng Jiang

**Affiliations:** 1 School of Integrated Circuits, Jiangnan University, Wuxi 214401, China; lishaomin22@jiangnan.edu.cn (S.L.); 6253803001@stu.jiangnan.edu.cn (Y.C.); 6253803005@stu.jiangnan.edu.cn (K.Q.); 2 China Resources Microelectronics Limited, Wuxi 214401, China; 3 Jiangsu Provincial Key Laboratory of MEMS Sensors and ASIC Manufacturing Technology, Wuxi 214401, China; 4 The 58th Research Institute of China Electronics Technology Group Corporation and National Key Laboratory of Integrated Circuits and Microsystems, Wuxi 214401, China; cetcweiyidan@163.com

**Keywords:** spintronic devices, spin-orbit torque, rare earth material, artificial synapses

## Abstract

Spin–orbit torque (SOT)-based spintronic devices have emerged as a preferred candidate for next-generation artificial synaptic devices due to their advantages of non-volatility, high speed, and low power consumption. The development of high-performance SOT-based artificial synaptic devices relies on the breakthrough in SOT-driven magnetization switching, wherein the performance regulation and structural design of the magnetic layer are the core critical factors. In this work, the Co/Ho multilayer system is employed as the magnetic layer to investigate its SOT-driven magnetization switching characteristics and application potential in artificial synapses. By regulating the periodic parameters of the Co/Ho multilayer structure, high perpendicular magnetic anisotropy (PMA) can be stably maintained in devices with relatively thick ferrimagnetic layers. Moreover, we elucidate the role of the antiferromagnetic coupling interface between Co and Ho in the multilayer structure in enhancing SOT efficiency and demonstrate the achievement of a high spin Hall angle of up to 0.22. The high SOT efficiency of the system enables it to drive the 8.4 nm-thick magnetic layer to achieve highly stable magnetization switching. Multistate magnetization switching behavior is observed, which can be used to simulate synaptic weight updates in neuromorphic networks, demonstrating the broad application prospects of this system in the field of artificial neural networks.

## 1. Introduction

Artificial intelligence (AI)-driven digital transformation of modern society requires high-efficiency computing technologies, with innovative high-density artificial synaptic and neuronal devices addressing the computational demands of complex tasks in the big data era [1,2]. Spintronic devices with non-volatility exhibit multistate storage behavior, among which those based on spin–orbit torque (SOT) are regarded as ideal candidates for constructing next-generation artificial synaptic devices, owing to their high-speed response and low-power consumption characteristics [3,4].

To fully exploit the application potential of SOT in artificial synaptic devices, high integration density and high stability are identified as the core performance requirements for SOT-based devices. Perpendicular magnetic anisotropy (PMA) is a critical prerequisite for achieving high device integration in SOT-based spintronic devices [5]. The SOT-driven perpendicular magnetization switching is usually studied in nonmagnetic/ferromagnetic (NM/FM) bilayers [6,7]. Excellent PMA characteristics are typically observed in devices with thinner ferromagnetic layers [8]. However, a thinner ferromagnetic layer compromises the thermal stability of the device [9]. Consequently, an irreconcilable trade-off emerges between the high integration density and high thermal stability of the device. Researchers have adopted various approaches to mitigate this trade-off. Ohtake et al. investigated the effect of substrate temperature on the film structure and magnetic properties, fabricating alloys with a highly ordered L_10_ phase, such as FePt and CoPt [10]. These alloy layers can achieve high perpendicular magnetic anisotropy with a thickness of 40 nm. Liu et al. prepared [Co/Ni]_n_ multilayer structures and observed stable perpendicular magnetization switching in the repeatedly stacked interfacial structure. Nevertheless, these systems still suffer from limitations in terms of tunability or compatibility [11].

In recent years, rare earth (RE)-transition metal (TM)-based ferrimagnetic systems have garnered extensive attention owing to their antiferromagnetically coupled sublattices and nearly zero net magnetization [12,13,14]. Utilizing the alloy layers formed by RE and TM as magnetization switching layers not only exhibits fast magnetization dynamics but also yields an additional enhancement in SOT efficiency [15,16]. Compared with RE-TM alloys system, RE/TM multilayer structures fabricated via alternating growth exhibit distinct advantages in terms of PMA characteristics [17,18]. Furthermore, rational interface engineering imparts these multilayer structures with superior tunability and flexibility in tailoring magnetic properties and spin transport characteristics [19,20]. To date, SOT-induced multistate magnetization switching behavior has been observed in RE-TM alloy systems, providing a critical physical foundation for artificial synaptic device development [21,22]. However, this highly promising multistate magnetization switching behavior has not been investigated in RE/TM multilayer systems, which possesses a higher degree of regulatory freedom and more prominent interfacial effects. Therefore, systematically investigating the modulation rules in RE/TM multilayer systems not only improves the magnetodynamics and SOT modulation theories but also supports high-tunability, high-integration, and low-power artificial synaptic device development.

In this work, we focus on the Co/Ho multilayer system, systematically investigating the SOT-driven perpendicular magnetization switching behavior and its application potential in artificial synapses. By tuning the periodicity of Co/Ho multilayers to optimize their magnetic properties and spin transport efficiency, the enhancement mechanism underlying the regulation of Co/Ho interfacial coupling on SOT efficiency is clarified. Furthermore, highly stable current-induced magnetization switching is achieved, on the basis of which the multilevel storage capability and the synaptic plasticity behavior are emulated. This work provides key theoretical insights and technical support for spintronic artificial synaptic device development, advancing neuromorphic computing toward low-power, high-integration operation.

## 2. Experimental Methods

### 2.1. Preparation Methods

**Figure 1:**
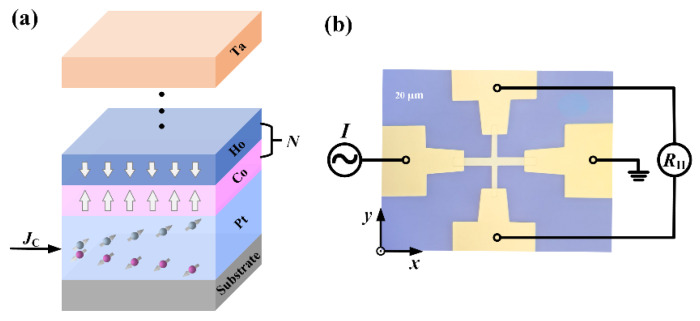
(**a**) Schematic of the stack of Ti/Pt/[Co (0.65)/Ho (0.55)]*_N_*/Ta multilayer. The white arrows represent the direction of the magnetic moment. (**b**) Optical image of the Hall ball device with schematic of Hall resistance measurement configuration.

The thin film stacks of Ti (2)/Pt (5)/[Co (0.65)/Ho (0.55)]*_N_*/Ta (2) were deposited on a thermally oxidized Si substrate using magnetron sputtering technique at base pressure of 3.0 × 10^−8^ Torr, as shown in Figure 1a. The numbers in parentheses are nominal thicknesses in nanometers, and *N* is the periodic number of the Co/Ho stacks. To ensure good PMA characteristics, parameter *N* is selected to be greater than 3. The Ti (2)/Pt (5)/Co (1.3)/Ta (2) structure is used as a reference sample, with *N* assigned a value of 0. The bottom 2 nm Ti layer is used for the adhesion layer, and the 5 nm Pt is used as the spin source. The top 2 nm Ta as the capping layer exhibits opposite spin Hall angle with Pt, synergistically enhancing the SOT efficiency of the whole system. The films were processed into Hall bar devices with the size of 20 × 200 μm using photolithography and ion milling techniques for electrical transport testing. Figure 1b shows the optical image of the patterned device.

### 2.2. Measurement Method

The magnetic hysteresis loops of the Co/Ho multilayer samples were measured via a vibrating sample magnetometer (VSM) to obtain the magnetic properties of the thin-film samples. Anomalous Hall resistance (*R*_H_) was collected through electrical transport measurements, and the anomalous Hall loops (*R*_H_-*H*_z_) were plotted to extract the magnetic properties of the patterned devices. The perpendicular magnetic anisotropy field (*H*_k_) was quantitatively derived by exploiting the dependence of the *R*_H_ on the in-plane magnetic field (*H*_x_). The second-harmonic Hall voltage measurement method was employed to acquire the first-harmonic (*V*_1ω_) and second-harmonic (*V*_2ω_) Hall voltage signals under different current densities, through which the effective fields of SOT, including the damping-like effective field (*H*_DL_) and field-like effective field (*H*_FL_), were quantitatively characterized. Further, the spin Hall angle(*θ*_SH_) of the devices was extracted via data fitting. Current-induced magnetization switching measurements were conducted to investigate the SOT-driven magnetization switching behaviors in the Co/Ho multilayer systems, with further demonstration of its multistate magnetization switching and synaptic plasticity.

## 3. Results and Discussion

### 3.1. Magnetic Properties of Co/Ho Multilayer Systems

**Figure 2:**
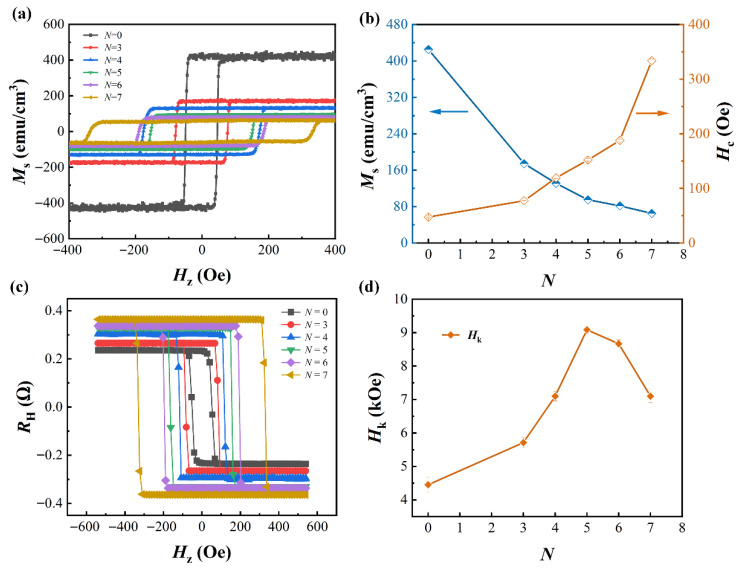
(**a**) Out-of-plane hysteresis loops of Co/Ho multilayer films. (**b**) Relationship between the saturation magnetization *M*_s_ and coercivity *H*_c_ with periods *N*. (**c**) *R*_H_ against *H*_z_ for the Hall bar devices with varying repetition numbers *N*. (**d**) Relationship between *H*_k_ and periods *N* for Co/Ho multilayer devices.

The out-of-plane magnetic hysteresis loops in the low magnetic field region were characterized by VSM at room temperature, as shown in Figure 2a. The well-defined rectangular magnetic hysteresis loops indicate that all samples possess excellent PMA characteristics. With the increase in the number of periods *N*, the multilayer samples still exhibit consistent switching behavior identical to that of the single Co layer. This is attributed to the strong interlayer coupling between the Ho and Co layers, which enables coherent magnetization switching under the assistance of an external magnetic field [23]. Figure 2b shows the saturation magnetization (*M*_s_) and coercive field (*H*_c_) of all samples, which were extracted from the out-of-plane magnetic hysteresis loops. As the number of periods *N* increases, the *M*_s_ exhibits a continuous decreasing trend, while the *H*_c_ shows a gradual increasing tendency. As an RE element, Ho possesses a magnetic moment antiparallel to that of Co. With the increase in the number of stacks, the enhanced antiferromagnetic exchange coupling effect between Co and Ho atoms is primarily responsible for the continuous reduction in *M*_s_ [24]. The variation in the *H*_c_ is relatively complex. The antiferromagnetically coupled interface between Co and Ho layers can enhance *H*_c_. Additionally, as the number of periods *N* increases, the gradual increase in Co layer thickness also contributes to the rise in the *H*_c_.

After applying a perpendicular magnetic field *H*_z_, the measured anomalous Hall resistance *R*_H_-*H*_z_ loops of the patterned samples are presented in Figure 2c, which exhibits a sharp magnetization reversal process consistent with that of the thin-film samples. To quantitatively characterize the PMA characteristics of the Hall bar devices, the perpendicular magnetic anisotropy field *H*_k_ was extracted based on the dependence of the *R*_H_ on the in-plane magnetic field *H*_x_. This method enables more efficient quantitative determination of *H*_k_ for Hall bar devices and has been widely adopted and reported in numerous studies [25,26]. Figure 2d shows the dependence of the *H*_k_ on the number of periods *N*. As the number of periods *N* increases, *H*_k_ exhibits a trend of initial increase followed by subsequent decrease. This is attributed to the intensified atomic intermixing between the ultrathin Co and Ho layers during the process of increasing alternating sputtering cycles [27,28]. At low sputtering cycles, the prominent interfacial anisotropy favors the enhancement of *H*_k_ for the system. However, with a further increase in sputtering cycles, the Co/Ho multilayer structure tends to form an alloy phase. The progressively blurred interfacial structure competes with the volume anisotropy induced by the formed alloy layers, resulting in the sample with a period number *N* = 5 exhibiting a relatively high *H*_k_ value of approximately 9 kOe, which is twice that of the reference sample.

### 3.2. Quantitative Analysis of SOT Efficiency in Co/Ho Multilayer Systems

**Figure 3:**
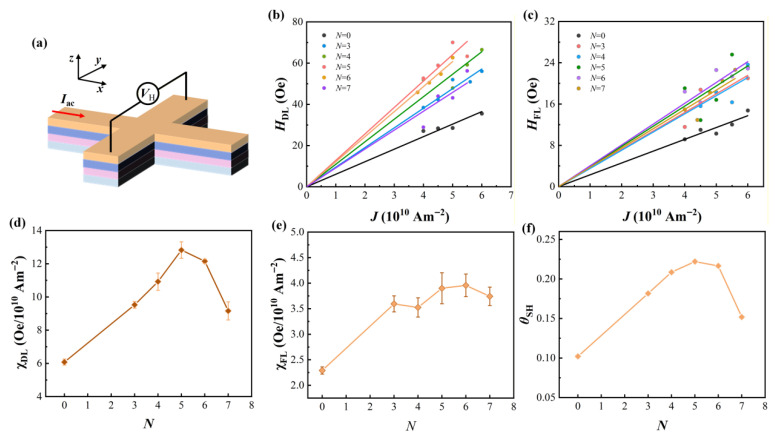
(**a**) Schematic of the harmonic measurement setup of the Hall bar devices. (**b**) *H*_DL_ and (**c**) *H*_FL_ as a function of the applied current density *J*. Colored scatter points are experimental data, and the solid lines are linear fits constrained to pass through the origin, from which the *χ*_DL_ and *χ*_FL_ are obtained. (**d**) *χ*_DL_ and (**e**) *χ*_FL_ as a function of the periods *N*. (**f**) Relationship between spin Hall angle *θ*_SH_ and periods *N*.

To quantitatively analyze the SOT efficiency of samples with different period numbers *N*, the second-harmonic measurement method was employed. The measurements were performed using an alternating current (*I*_ac_) source and a lock-in amplifier operating at a frequency of 307 Hz to detect the Hall voltage (*V*_H_). Figure 3a presents a schematic diagram of the harmonic Hall voltage measurement setup. During the measurements, sweeping magnetic fields *H*_x_ and *H*_y_ were applied parallel and perpendicular to the current direction, respectively, to extract the *V*_1ω_ and *V*_2ω_ of the samples under different current density excitation conditions. The *H*_DL_ and *H*_FL_ were derived from the Hall voltage measurement data using the following equations:

$$\begin{array}{c} H_{DL\left(FL\right)}=-2\left(\frac{{dV}_{2w}}{{dH}_{x\left(y\right)}}\right)/\left(\frac{d^{2}V_{1w}}{{dH}_{x\left(y\right)}^{2}}\right). \end{array}$$

Figure 3b,c show the *H*_DL_ and *H*_FL_ obtained by sweeping the transverse magnetic field *H*_x_ and longitudinal magnetic field *H*_y_ under different current densities *J*, respectively. It can be observed that the effective *H*_DL_ maintains a good linear relationship with the current density. However, the effective *H*_FL_ data obtained at the same current density are relatively scattered, indicating that the extracted effective *H*_FL_ of the samples is relatively weak. Figure 3d,e show the relationships between the extracted effective damping-like field per unit current density (*χ*_DL_), effective field-like field per unit current density (*χ*_FL_), and the period number *N* of the samples, respectively. For the Co/Ho multilayer structures, *χ*_DL_ exhibits a trend of first increasing and then decreasing as the period number *N* increases. When the number of alternately deposited Co/Ho interfaces reaches 5, the SOT efficiency is more than twice that of the reference sample, peaking at 12.8 ± 0.49 Oe/10^10^ A·m^−2^. This indicates that an additional spin current is generated in the Co/Ho multilayer structures. The antiferromagnetic coupling effect between Co/Ho interfaces exerts a significant influence on the generation and transport of spin current, which constitutes the primary mechanism for the enhancement of SOT efficiency in the Co/Ho multilayer structures [29]. Nevertheless, with the continuous increase in period number *N*, *χ*_DL_ begins to show a decreasing trend. This trend is consistent with the variation in the perpendicular magnetic anisotropy field *H*_k_ as a function of period number *N*. This is attributed to the reason that repeated sputtering leads to the blurring of Co/Ho interfaces, causing the multilayer structures to gradually transform into alloy structures. As the interfacial characteristics disappear, the antiferromagnetic coupling effect between Co/Ho interfaces weakens progressively, and the spin transport and coupling mechanisms in the multilayer structures undergo a corresponding transformation. Compared with the CoHo multilayer systems, the spin current generated by the CoHo alloy layers is relatively low. This phenomenon has also been reported in previous studies on ferromagnetic rare-earth alloy layers [30]. In addition, compared with the reference sample, the presence of Co/Ho interfaces results in a significant enhancement of *χ*_FL_. Nevertheless, with the further increase in the period number *N*, *χ*_FL_ exhibits a less pronounced growth trend. By comparing Figure 3d,e, it can be seen that the values of *χ*_DL_ and *χ*_FL_ differ by nearly one order of magnitude, which indicates that *H*_DL_ has a more prominent impact on device performance, while the influence of *H*_FL_ is negligible.

To more accurately characterize the role of SOT in the magnetization switching process of this system, the expression for the *θ*_SH_ is thus revised as follows:

$$\begin{array}{c} \theta_{SH}=\frac{2e\mu_{0}M_{net}t_{f}}{\hbar }\frac{H_{DL}}{J} \end{array}$$

where *e* is the electron charge, *μ*_0_ is the permeability vacuum, *ħ* is the reduced Planck’s constant, *M*_net_ is the net saturation magnetization of the Co/Ho systems, and *t*_f_ is the thickness of the FM layer. Figure 3f illustrates the variation in the *θ*_SH_ with the period number *N* for the Co/Ho multilayer samples. As the period number *N* increases, the variation trend of *θ*_SH_ is consistent with that of *χ*_DL_. When the period number *N* = 5, the *θ*_SH_ reaches the maximum value of approximately 0.22, which is twice that of the reference sample. This value also exhibits a distinct advantage in comparison with those of other Pt-based structure samples.

### 3.3. Magnetization Switching Characteristics and Stability Analysis

**Figure 4:**
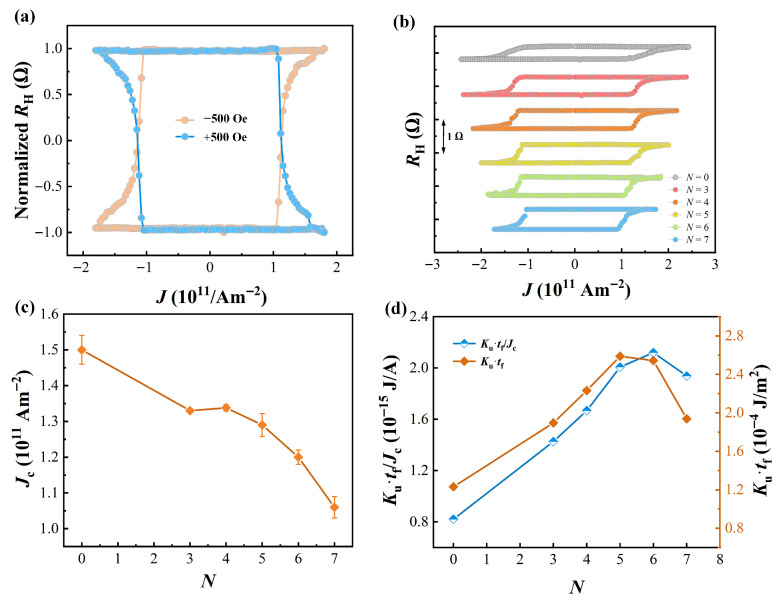
(**a**) Pt/[Co/Ho]_6_ device under an in-plane field *H*_x_ of ±500 Oe. (**b**) Magnetization switching loops for Co/Ho multilayer devices with different periods *N* under −500 Oe. (**c**) Relationship between critical switching current density *J*_c_ and periods *N*. (**d**) *K*_u_·*t*_f_/*J*_c_ and *K*_u_·*t*_f_ as a function of periods *N* in the Co/Ho multilayer systems.

To investigate the impact of the periods *N* on the SOT-driven switching behavior in Co/Ho multilayer systems, current-induced magnetization switching measurements were conducted. Pulses of 35 mA with a width of 5 ms were applied to the Hall cross device. A fixed magnetic field was applied while scanning the writing pulse current along the x-direction. Another small current pulse of 200 μA was employed to read the Hall resistance. Figure 4a shows the normalized *R*_H_ of the sample with periods *N* of 6 under *H*_x_ = ±500 Oe. *R*_H_ as a function of the pulsed current density *J* reveals a clear transition between two resistance states. Applying *H*_x_ in different directions results in switching loops with opposite polarities, which is a typical characteristic of SOT-induced magnetization switching. +*H*_x_ corresponds to a clockwise cycle, while −*H*_x_ corresponds to a counterclockwise cycle. Figure 4b shows the current-induced magnetization switching loops of the devices with different *N* value under external in-plane magnetic field of −500 Oe. All samples can realize efficient magnetization switching with a switching ratio larger than 90% under *H*_x_ = −500 Oe. Figure 4c shows the *N*-dependent critical current density (*J*_c_). Here, *J*_c_ is identified as the critical current density for 50% switching of magnetization state. With the increase in the number of Co/Ho multilayer stacks, the *J*_c_ exhibits a gradual decreasing trend. When *N* = 7, the *J*_c_ for driving magnetization switching is 1.1 × 10^11^ A·m^−2^, which is 1.5 times lower than that of the reference sample. Although *J*_c_ is comparable to that required for magnetization switching of a 1.3 nm ferromagnetic layer driven by Pt-based structures, it is important to emphasize that the free layer thickness of the Pt/[Co/Ho]_7_ sample reaches 8.4 nm. Typically, the increase in magnetic layer thickness tends to result in a rise in the critical switching current. In contrast, the Co/Ho multilayer structure can maintain a relatively low *J*_c_ while retaining a large free layer thickness, demonstrating significant advantages in magnetization switching performance.

The thermal stability factor (Δ) is typically used to evaluate the storage lifetime of devices, where a higher Δ can ensure stable switching of devices for more than a decade. At room temperature, when the effective area of devices is consistent, the Δ can be assessed by the product of the magnetic anisotropy constant (*K*_u_) and the thickness (*t*_f_) of the magnetic layer, where *K*_u_ = *H*_k_*M*_s_/2 represents the effective vertical anisotropy energy density. For the Co/Ho multilayer samples with different stack numbers *N*, it is noted that the *J*_c_ varies with the increase in *t*_f_. To provide a more equitable comparison condition, the switching current density is used to normalize the difference in energy barrier under different cycle numbers. Specifically, *K*_u_·*t*_f_/*J*_c_ is adopted to characterize the magnetization switching stability of the Co/Ho multilayer samples. Figure 4d presents the variations in *K*_u_·*t*_f_/*J*_c_ and *K*_u_·*t*_f_ with the periods *N* in the Co/Ho multilayer samples. With the increase in *N*, both *K*_u_·*t*_f_/*J*_c_ and *K*_u_·*t*_f_ are significantly enhanced compared to the reference sample, indicating the improved magnetization switching stability of the multilayer samples. Furthermore, with a further increase in *N*, both *K*_u_·*t*_f_/*J*_c_ and *K*_u_·*t*_f_ exhibit a decreasing trend, which also confirms that the Co/Ho multilayer structure gradually transitions to an alloy phase. Eventually, the Pt/[Co/Ho]_6_ sample demonstrates the highest switching stability.

### 3.4. Multistate Magnetization Switching and Synaptic Plasticity Simulation

**Figure 5:**
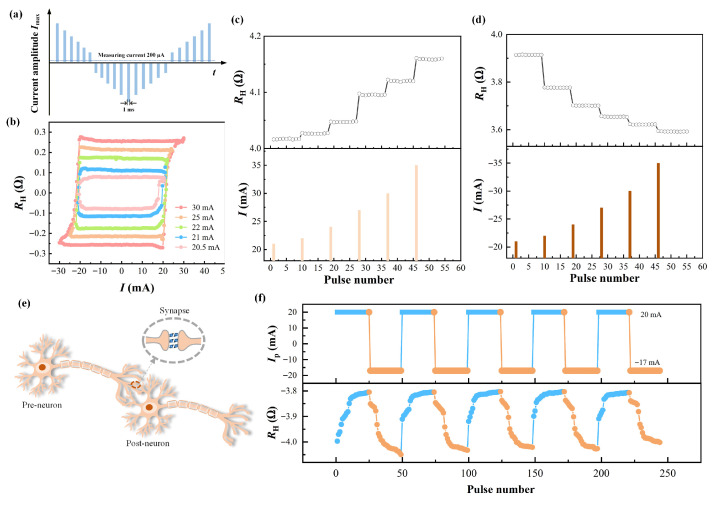
(**a**) Schematic of the measurement procedure for pulse current-induced magnetization switching. (**b**) Magnetization switching loops of the Pt/[Co/Ho]_6_ device under different *I*_max_. Response of the Hall resistance *R*_H_ of the Pt/[Co/Ho]_6_ device to current pulses number with gradually increasing amplitude of (**c**) positive current pulse and (**d**) negative current pulse. (**e**) Schematic of the artificial neurons and synapses constructed by spintronic devices. (**f**) Response of the Hall resistance *R*_H_ of the Pt/[Co/Ho]_6_ device under alternating positive (20 mA) and negative (−17 mA) current pulses.

Co/Ho multilayer samples exhibit excellent performance characteristics during the magnetization switching process. To further verify their application potential in the field of multistate memory, we applied a series of current pulses ranging from 20.5 to 30 mA with different maximum magnitudes (*I*_max_) to the Pt/[Co/Ho]_6_ sample, and the measurement process is illustrated in Figure 5a. Figure 5b shows the switching loops with the monotonic increasing switching ratio under different *I*_max_ values. Different *I*_max_ values resulted in various *R*_H_ values, indicating the potential to modulate the magnetization state by varying the current density. This also confirms the multistate storage behavior during the current-induced magnetization switching process in the ferrimagnetic Co/Ho multilayer devices.

Next, we demonstrate the continuous manipulation of magnetization state by current pulses. In the experiment, a series of unipolar current pulses with different amplitudes were applied to the sample under *H*_x_ = −500 Oe, and the *R*_H_ values were recorded nine consecutive times using a 1 mA test current after the application of each pulse. With the increasing amplitude of positive (negative) current pulse, *R*_H_ value increases (decreases), as shown in Figure 5c,d. This result further demonstrates that effective manipulation of the magnetization state in Co/Ho multilayer samples can be achieved by precisely controlling the amplitude and polarity of pulse currents, thereby enabling the realization of a more reliable multistate memory function. Furthermore, the *R*_H_ stabilizes at a constant value between the two pulse widths, demonstrating that the Pt/[Co/Ho]_6_ sample exhibits favorable data stability for application as a multistate memory device.

The current-induced resistance modulation capability in the Pt/[Co/Ho]_6_ sample endows it with corresponding advantages for the fabrication of artificial synaptic devices. In biomimetic neural systems, the neuromorphic networks comprise neurons and synapses. These synapses connect two neurons and modulate the connection strength within the Hall bar device by adjusting their weights. In this process, alterations in synaptic weights occur concomitantly, leading to the generation of excitatory postsynaptic potentials (EPSP) or inhibitory postsynaptic potentials (IPSP). As external stimuli change, synaptic weights undergo corresponding adjustments, thereby mediating learning and memory behaviors in neural networks. The schematic diagram of the structure of an artificial synapse is presented in Figure 5e.

The variations in Hall resistance, which are continuously tuned by programming consecutive pulse sequences, are desired to imitate synaptic behavior. The magnetization of the Pt/[Co/Ho]_6_ device was switched to the downward-magnetized state. During the measurement, an external auxiliary magnetic field of −500 Oe was fixed, and 250 cycles of positive-negative alternating current pulses were continuously applied to the device. The positive and negative current pulses were set to amplitudes of 20 mA and −17 mA, respectively, with a pulse width of 1 ms for both polarities. The corresponding schematic of the pulse current and the response of the *R*_H_ to the applied current pulses are presented in Figure 5f. Notably, *R*_H_ varied correspondingly with the change in the applied current pulses, demonstrating the realization of artificial synaptic functionality. Specifically, when positive current pulses were applied to the device, the *R*_H_ value increased with the number of positive pulses, which corresponds to the EPSP behavior of synaptic plasticity. In contrast, when negative current pulses were applied, the *R*_H_ value decreased with the number of negative pulses, which is analogous to the IPSP behavior of synaptic plasticity. These results demonstrate the feasibility of the Pt/[Co/Ho]_6_ device for emulating synaptic plasticity. Notably, asymmetric behavior between the EPSP and IPSP has been observed, which arises from differences in the domain wall (DW) propagation velocity under positive and negative current pulses [31]. The ferrimagnetic Co/Ho multilayer devices manifest SOT-driven polymorphic magnetic switching behavior and synaptic characteristics, presenting promising applications in multilevel storage and artificial neuromorphic computing.

## 4. Conclusions

In conclusion, we systematically investigate the SOT-driven magnetization switching characteristics of Co/Ho multilayer systems and clarify their application values in artificial synaptic devices. By regulating the periodicity of Co/Ho multilayer systems, devices with relatively thick ferrimagnetic layers can stably maintain high PMA characteristic, with a maximum perpendicular magnetic anisotropy field of 9 kOe. Quantitative analysis of the variation in SOT efficiency with the number of *N* reveals that the antiferromagnetic coupling at the Co/Ho interface is the key to enhancing SOT efficiency. Current-induced magnetization switching tests demonstrate that the high SOT efficiency of the system can drive stable magnetization switching of an 8.4 nm-thick ferromagnetic layer, with a switching current density as low as 1.1 × 10^11^ A·m^−2^. Performance comparison with other ferromagnetic layers verifies the significant advantages of the Co/Ho multilayer structure as a magnetic layer. In addition, based on the performance of the Pt/[Co/Ho]_6_ device, it is confirmed that the Co/Ho multilayer system exhibits multistate magnetization switching capability. Combined with synaptic plasticity simulations, this further demonstrates the great application potential of the high-stability magnetization switching system in artificial neural networks.

## Data Availability

The original contributions presented in this study are included in the article. Further inquiries can be directed to the corresponding authors.
